# Mortality predictors and diagnostic challenges in adult tuberculous meningitis: a retrospective cohort of 100 patients

**DOI:** 10.1186/s41182-025-00738-0

**Published:** 2025-04-24

**Authors:** Mahboubeh Maleki Rad, Mahboubeh Haddad, Fereshte Sheybani, Matin Shirazinia, Maliheh Dadgarmoghaddam

**Affiliations:** 1 Department of Infectious Diseases and Tropical Medicine, Faculty of Medicine, Mashhad University of Medical Sciences, Mashhad, Iran; 2 Faculty of Medicine, Mashhad University of Medical Sciences, Mashhad, Iran; 3 Department of Community Medicine, Faculty of Medicine, Mashhad University of Medical Sciences, Mashhad, Iran

**Keywords:** Tuberculosis meningitis, Lancet scoring system, Survival, Cohort, Anti-tuberculous medications, All-cause mortality, Diagnostic certainty

## Abstract

**Objective:**

This study aimed to assess the clinical characteristics, diagnostic certainty, outcomes, and predictors of mortality in patients diagnosed with tuberculous meningitis (TBM), using the Lancet scoring system for diagnostic certainty.

**Methods:**

A retrospective cohort was conducted on 100 patients diagnosed with TBM. Patients were classified based on the Lancet scoring system into definite, probable, and possible TBM categories. Clinical features, neuroimaging findings, cerebrospinal fluid (CSF) analysis, and outcomes were analyzed.

**Results:**

The median age of patients was 36.5 years, with 57.0% male. The most common symptoms were fever (64.7%), headache (63.6%), and altered consciousness (60.0%). Hydrocephalus was present in 40.0% of cases. Diagnosis certainty was classified as possible (63.0%), probable (22.0%), and definite (15.0%). The in-hospital mortality rate was 18.0%, with 12-month survival rates of 69.7%. There was no significant difference between the level of diagnostic certainty and the survival of patients. Significant predictors of mortality included hydrocephalus (hazard ratio [HR]: 3.65, 95% CI 1.67 to 7.97), hemoglobin levels (HR: 0.75, 95% CI 0.64 to 0.89), age (HR: 1.04, 95% CI 1.02 to 1.06), CSF pleocytosis (HR: 0.34, 95% CI 0.14 to 0.84), and altered consciousness at admission (HR: 19.23, 95% CI 2.57 to 143.85).

**Conclusion:**

TBM remains a critical concern with significant mortality and morbidity. Key predictors of mortality, including altered consciousness, hydrocephalus, and older age, highlight the need for early detection and tailored interventions. In most cases, the diagnosis cannot be definitively confirmed and is instead categorized as probable or possible. Our study demonstrates that survival rates were comparable across definite, probable, and possible TBM categories, supporting the value of empirical treatment when definitive confirmation is not feasible.

## Introduction

*Mycobacterium tuberculosis* primarily infects the lungs, but central nervous system (CNS) involvement, particularly tuberculous meningitis (TBM), is the most severe manifestation, accounting for approximately 1% of all tuberculosis (TB) cases globally [1, 2]. A recent modeling study estimated that in 2019, approximately 164,000 adults developed TBM worldwide, with a case fatality rate of 48% [3]. The incidence and outcomes of TBM are notably influenced by HIV co-infection. A systematic review and meta-analysis reported that among adults living with HIV, the prevalence of TBM was 13.6%, with an incidence rate of 1.5 per 1000 person-years, and a case fatality rate of 38.1% [4].

Delayed diagnosis of TBM remains a major challenge due to nonspecific symptoms and the low sensitivity of conventional diagnostic methods [5, 6]. Consequently, it is often misdiagnosed or diagnosed too late, contributing to poorer clinical outcomes and higher mortality [7–9]. A study analyzing microbiological diagnosis and mortality in TBM patients found an overall case fatality rate of 20.42%, highlighting the critical need for early and accurate diagnostic approaches [10].

To address these issues, researchers and expert groups have worked to develop standardized criteria for the early diagnosis of TBM. The diagnostic framework proposed by Thwaites et al. [11] integrates clinical and laboratory features, demonstrating the benefits of a multifaceted diagnostic approach. This method has proven especially valuable in resource-limited settings, where access to advanced diagnostic tools may be restricted. Additionally, Marais et al. provided a consensus case definition in *Lancet Infectious Diseases* aimed at creating a uniform diagnostic framework that can be applied across various settings and patient populations. This standardization enhances diagnostic accuracy and facilitates better comparability of research findings, ultimately improving clinical management of TBM [12]. These efforts to define and implement standardized diagnostic criteria are essential for overcoming the challenges of TBM diagnosis and ensuring timely and effective treatment.

Due to the suboptimal sensitivity of diagnostic tests for TBM, it is common practice to initiate empirical treatment with anti-TB medications in suspected cases after excluding other potential diagnoses [13]. This strategy aims to provide prompt treatment despite uncertain diagnostic confirmation. In this study, we aimed to describe the clinical characteristics, laboratory and imaging findings, long-term survival, and predictors of mortality in patients with TBM. Additionally, we evaluated the survival probabilities among patients with definitive, probable, and possible diagnoses of TBM who received anti-TB treatment.

## Methods

This retrospective cohort study was conducted at two main referral centers for CNS infections in Mashhad, Iran, the country's second-largest city in the northeast. Mashhad is the capital city of Khorasan Razavi province. The study included individuals aged 15 and older who were diagnosed with TBM between March 21, 2016, and March 20, 2022. Data were collected using a standardized checklist to record patient information, including age, gender, clinical features, radiological and laboratory findings, and clinical outcomes. For patients admitted between March 2016 and September 2019, medical records and discharge letters were reviewed. Data for those admitted between October 2019 and March 2022, who were part of our registry of community-acquired suspected CNS infections, were prospectively collected using an online patient registration form. The transition from retrospective (March 2016–September 2019) to prospective (October 2019–March 2022) data collection occurred because our registry for community-acquired suspected CNS infections was implemented in October 2019, allowing for structured prospective data entry from that point onward.

### The level of diagnostic certainty for TBM (based on the Lancet scoring system) [12]

The clinical criteria for diagnosing TBM included symptoms and signs of meningitis, such as headache, restlessness, vomiting, fever, neck stiffness, seizures, focal neurological deficits, altered consciousness, or lethargy. Patients were categorized as having definite, probable, or possible TBM based on the diagnostic scoring system proposed by Marais et al. [11].

Definite TBM was diagnosed in patients who met the clinical criteria and had either a positive CSF smear for acid-fast bacilli, a positive CSF culture for M. tuberculosis, or a positive PCR result for M. tuberculosis. Alternatively, definite TBM was also assigned when acid-fast bacilli were identified in the background of histological changes consistent with TB in the brain or spinal cord, accompanied by clinical symptoms and CSF inflammatory changes or evidence of meningitis at autopsy.

Probable TBM was defined in patients who met the clinical criteria and had a diagnostic score of 10 or more when brain imaging was not available, or 12 or more when brain imaging was performed, in addition to the exclusion of alternative diagnoses. Possible TBM was defined as meeting the clinical criteria with a diagnostic score of 6–9 without brain imaging, or 6–11 with brain imaging, again contingent upon ruling out other causes. It should be noted that lumbar puncture and brain imaging were mandatory for establishing diagnostic certainty in all cases.

Cases were excluded if an alternative confirmed diagnosis explained the patient's clinical presentation. This was defined as a diagnosis other than TBM in a confirmed patient, with no evidence supporting concurrent TBM or two simultaneous diseases [12]. All patients who met the criteria for definite, probable, or possible TBM were included in the study.

Exclusion criteria involved the presence of an alternative confirmed diagnosis that could explain the patient's clinical presentation. The exclusion of alternative causes of meningitis was based on a structured diagnostic algorithm previously described in our prior study [14]. This algorithm incorporated a stepwise evaluation of clinical features, laboratory data, and neuroimaging findings. All patients underwent brain imaging (MRI or CT) prior to lumbar puncture to assess for meningeal enhancement, space-occupying lesions, infarctions, or hydrocephalus. Cerebrospinal fluid (CSF) analysis included cell counts, protein and glucose levels, Gram staining, and fungal and bacterial cultures.

Additional diagnostic tests were performed based on clinical suspicion and risk factors, including CSF and serum testing for *M. tuberculosis* (AFB smear, culture, PCR), Cryptococcus (CSF cryptococcal antigen), Brucella (serum and CSF Wright and 2ME tests in endemic regions), syphilis (CSF VDRL), HIV (serology), and other pathogens. Autoimmune and paraneoplastic evaluations such as CSF cytology, flow cytometry, ANA profile, anti-dsDNA, IgG4, CSF ACE, HLA-B51, Pathergy test, PR3-ANCA, and MPO-ANCA were performed if indicated. In diagnostically challenging cases, advanced imaging like PET-CT or CT scans, and occasionally leptomeningeal or brain biopsies, were utilized.

Patients classified as probable or possible TBM were only included after exclusion of alternative diagnoses using the aforementioned algorithm. All available microbiological results, including CSF AFB smear, culture, and nucleic acid amplification tests (NAATs) for M. tuberculosis, were reviewed. However, due to resource limitations, AFB smear was the most commonly performed test, with CSF cultures not routinely available, particularly in probable and possible TBM cases.

At our centers, the standard practice for managing suspected TBM involves the empirical initiation of anti-TB therapy during the diagnostic evaluation. If no alternative cause for the meningitis is found and TBM remains a concern, the anti-TB treatment is continued. In cases where treatment was not started initially, it is promptly initiated, with careful monitoring of the patient’s response to ensure effective management.

Patients were classified as having probable or possible TBM only if no alternative diagnosis was established through this algorithm. Empirical anti-TB therapy was initiated when TBM remained a strong concern, with treatment continuation based on clinical response and follow-up testing.

### Modified british medical research council (BMRC)

The British Medical Research Council (BMRC) initially developed a disease severity grading system for TBM in the 1948 streptomycin trial. With the introduction of the Glasgow Coma Scale (GCS) in 1974, the grading system was modified to categorize TBM severity as grade I (GCS 15, no focal deficits), grade II (GCS 11–14 or 15 with focal deficits), and grade III (GCS ≤ 10). This classification remains widely used for prognostic assessment, as advanced TBM (grade III) is associated with a significantly higher risk of mortality and long-term neurological impairment [15].

For patients who survived and were discharged, follow-up assessments were conducted by telephone at the end of the study to evaluate their survival and neuropsychosocial functioning based on self-reported outcomes.

#### Statistics

Continuous data were summarized using medians and percentile 25 to percentile 75, while categorical variables were described with frequencies and percentages. Survival analysis was performed using Kaplan–Meier curves, log-rank tests, and Cox regression. Since the event per variable of the analysis was 30, we reported only the univariable analysis (crude model) and the age-adjusted model, as age is an established risk factor for mortality among patients with TBM [16]. The constant hazard assumption was examined by testing, with a *P*-value less than 0.05 indicating a departure from the proportionality assumption. A *P*-value of less than 0.05 was considered statistically significant for all analyses.

#### Ethics

Informed consent was obtained from all patients or their legally authorized representatives. The study was approved by the ethics committee of Mashhad University of Medical Sciences under code 4/8/2025.

## Results

### Patient characteristics

A total of 100 patients diagnosed with TBM were included in the study. The median age of the patients was 36.5 years (percentile 25 to percentile 75, 27.5 to 55.0 years), with 13 (13.0%) being elderly (≥ 65 years) (Table 1). The majority of the patients were male (57.0%, 57).

**Table 1:** Demographics, Underlying Comorbidities, and Clinical Manifestations of Patients with Tuberculous Meningitis (N = 100)

Variables	Possible (n = 63)	Probable (n = 22)	Definite (n = 15)	Total (n = 100)
Age (years), median (percentile 25 to percentile 75)	38.0 (27.0 to 58.0)	33.5 (27.0 to 51.0)	38.0 (29.0 to 45.0)	36.5 (27.5 to 55.0)
Elderly (≥ 65 years), n (%)	8 (12.7)	2 (9.1)	3 (20.0)	13 (13.0)
Gender (male), n (%)	38 (60.3)	12 (54.6)	7 (46.7)	57 (57.0)
Underlying comorbidities, n (%)				
Immunocompromised	16/61 (26.2)	4 (18.2)	2 (13.3)	22/98 (22.5)
Cardiovascular disorders	10/60 (16.7)	3 (13.6)	2 (13.3)	15/97 (15.5)
Diabetes mellitus	8/61 (13.1)	1 (4.6)	0	9/98 (9.2)
Rheumatologic disorders	6/61 (9.8)	1 (4.6)	2 (13.3)	9/98 (9.2)
Cancer	1/61 (1.6)	0	0	1/98 (1.0)
HIV/AIDS	0	1 (4.6)	0	1/98 (1.0)
Addiction	13/56 (23.2)	3 (13.6)	3 (20.0)	19/93 (20.4)
Clinical manifestations, n (%)				
Headache	36/62 (58.1)	15 (68.2)	12 (80.0)	63/99 (63.6)
Fever	40/62 (64.5)	14 (63.6)	10 (66.7)	64/99 (64.7)
Seizures	6/62 (9.7)	5 (22.7)	2 (13.3)	13/99 (13.1)
Altered consciousness	34 (54.0)	15 (68.2)	11 (73.3)	60 (60.0)
Neck stiffness	27/62 (43.6)	12 (54.6)	5 (33.3)	44/99 (44.4)
FNDs	30/60 (50.0)	12 (54.6)	6/14 (42.9)	48/96 (50.0)
Visual problem	14/62 (22.6)	7 (31.8)	2 (13.3)	23/99 (23.2)
Speech problem	7/62 (12.0)	5 (22.7)	3 (20.0)	15/99 (15.2)
Paresis	20/62 (32.3)	8 (36.4)	5 (33.3)	33/99 (33.3)
Cranial nerve palsies	16/59 (27.1)	8 (36.4)	2 (14.3)	26/95 (27.4)
Classic triad of meningitis^1^	12/62 (19.4)	4 (18.2)	3 (20.0)	19/99 (19.2)
Symptom duration (days), median (percentile 25 to percentile 75)	10.0 (5.0 to 30.0), n = 56	14.0 (7.0 to 30.0)	21.0 (5.0 to 45.0)	12.0 (6.0 to 30.0)

HIV, human immunodeficiency virus; AIDS, acquired immunodeficiency syndrome; FNDs, focal neurologic deficits;

^1^Characterized by fever, altered consciousness, and neck stiffness

### Underlying comorbidities

Among the patients, 22.5% (22/98) were immunocompromised, 15.5% (15/97) had cardiovascular disorders, and 9.2% (9/98) had diabetes mellitus. Additionally, 1.0% (1/98) of the participants were diagnosed with HIV/AIDS, and 20.4% (19/93) had a history of substance addiction.

### Clinical manifestations

The most common clinical manifestations included fever (64.7%, 64/99) and headache (63.6%, 63/99). Altered consciousness was observed in 60.0% (60) of patients, while 44.4% (44/99) presented with neck stiffness. Focal neurologic deficits were noted in 50.0% (48/96) of the cases.

Cranial nerve paresis was observed in 26 out of 95 patients (27.4%), of whom 12 (46.2%) had multiple cranial nerve paresis, primarily affecting the abducent, oculomotor, and facial nerves. Other symptoms included visual problems (23.2%, 23/99), speech problems (15.2%, 15/99), and limb paresis (33.3%, 33/99). The classic triad of meningitis (fever, altered consciousness, and neck stiffness) was observed in 19.2% (19/99) of the patients.

### Neuroimaging findings

Neuroimaging revealed hydrocephalus in 40.0% (38/95) of patients and meningeal enhancement in 35.8% (34/95). Cerebral infarction was present in 28.4% (27/95), while tuberculomas or abscesses were noted in 6.3% (6/95) (Table 2).

**Table 2:** Neuroimaging, Laboratory Findings, and Disease Severity in Patients with Tuberculous Meningitis (N = 100)

Variables	Possible (n = 63)	Probable (n = 22)	Definite (n = 15)	Total (n = 100)
Neuroimaging findings, n (%)				
Normal	21/59 (35.6)	2 (9.1)	4/14 (28.6)	27/95 (28.4)
Hydrocephalus	20/59 (33.9)	11 (50.0)	7/14 (50.0)	38/95 (40.0)
Meningeal enhancement	16/59 (27.1)	13 (59.1)	5/14 (35.7)	34/95 (35.8)
Cerebral infarction	11/59 (18.6)	11 (50.0)	5/14 (35.7)	27/95 (28.4)
Tuberculoma/abscess	2/59 (3.4)	2 (9.1)	2/14 (14.3)	6/95 (6.3)
Laboratory features				
CSF leucocytes (per µl), median (percentile 25 to percentile 75)	130.0 (40.0 to 350.0), n = 53	66.0 (10.0 to 200.0)	110.0 (5.0 to 200.0)	110.0 (20.0 to 295.0), n = 90
CSF pleocytosis, n (%)	49/53 (92.5)	18 (81.8)	12 (80.0)	79/90 (87.8)
CSF polymorphonuclears (%), median (percentile 25 to percentile 75)	20.0 (5.0 to 50.0), n = 49	10.0 (5.0 to 30.0)	56.5 (10.0 to 85.0), n = 14	20.0 (5.0 to 65.0), n = 85
CSF lymphocyte predominance^1^, n (%)	36/49 (73.5)	17 (77.3)	7/14 (50.0)	60/85 (70.6)
CSF protein (mg/dl), median (percentile 25 to percentile 75)	138.0 (83.0 to 176.0), n = 54	118.0 (96.0 to 167.0), n = 21	91.1 (68.0 to 127.0), n = 14	122.0 (84.0 to 166.0), n = 89
CSF hyperproteinorrhachia, n (%)	47/54 (87.0)	20/21 (95.2)	14/14 (100.0)	81/89 (91.0)
CSF glucose (mg/dl), median (percentile 25 to percentile 75)	37.0 (21.0 to 58.0), n = 54	27.0 (19.0 to 37.0), n = 21	21.5 (15.0 to 43.0), n = 14	34.0 (19.0 to 49.0), n = 89
CSF hypoglycorrhachia, n (%)	30/54 (55.6)	17/21 (81.0)	10/14 (71.4)	57/89 (64.0)
Serum sodium (mg/dl), median (percentile 25 to percentile 75)	137.0 (134.0 to 140.0), n = 56	133.0 (131.0 to 136.0)	131.0 (130.0 to 137.0), n = 14	135.0 (131.0 to 140.0), n = 92
ESR (mm/hour), median (percentile 25 to percentile 75)	24.0 (10.0 to 40.0), n = 53	25.0 (10.0 to 43.0), n = 19	42.0 (20.0 to 57.0), n = 11	25.0 (10.0 to 44.0), n = 83
CRP (mg/L), median (percentile 25 to percentile 75)	18.8 (6.3 to 52.5), n = 49	12.6 (2.2 to 70.5), n = 16	32.7 (16.4 to 81.1), n = 10	18.8 (5.6 to 53.9), n = 75
Leukocytes (× 10^3^/per µl), median (percentile 25 to percentile 75)	8.9 (7.3 to 10.6), n = 57	8.1 (5.8 to 10.2)	8.7 (7.3 to 12.5), n = 14	8.8 (7.0 to 10.6), n = 93
Hemoglobin (mg/dL), median (percentile 25 to percentile 75)	13.5 (11.6 to 14.3), n = 57	12.3 (9.3 to 13.5), n = 21	12.9 (11.3 to 13.7), n = 14	13.0 (11.3 to 14.2), n = 92
Platelets (× 10^3^/per µl), median (percentile 25 to percentile 75)	246.5 (198.0 to 317.0), n = 56	249.0 (187.0 to 316.0)	242.5 (214.0 to 302.0), n = 14	245.0 (195.0 to 307.5), n = 92
Modified BMRC severity stage, n (%)				
I	12 (19.1)	2 (9.1)	3 (20.0)	17 (17.0)
II	37 (58.7)	18 (81.8)	8 (53.3)	63 (63.0)
III	14 (22.2)	2 (9.1)	4 (26.7)	20 (20.0)
Length of hospital stay (days), median (percentile 25 to percentile 75)	19.0 (10.0 to 31.0)	21.0 (13.0 to 27.5), n = 20	23.0 (11.0 to 31.0)	20.0 (11.0 to 30.0), n = 98

CSF, cerebrospinal fluid; ESR, erythrocyte sedimentation rate; CRP, C-reactive protein; BMRC, British Medical Research Council

^1^Defined as comprising more than 50 percent lymphocytes in cerebrospinal fluid

### Laboratory features

Cerebrospinal fluid (CSF) analysis showed pleocytosis in 87.8% (79/90) of patients, with a lymphocyte predominance in 70.6% (60/85). CSF hypoglycorrhachia was observed in 64.0% (57/89) of cases and CSF hyperproteinorrhachia was found in 91.0% (81/89).

The median CSF protein level was 122.0 mg/dL (percentile 25 to percentile 75, 84.0 to 166.0 mg/dL), and the median CSF glucose level was 34.0 mg/dL (percentile 25 to percentile 75, 19.0 to 49.0 mg/dL).

### Diagnosis and hospitalization

The level of certainty for the diagnosis of TBM was classified as possible in 63.0% (63), probable in 22.0% [21], and definite in 15.0% [14]. The median length of hospital stay was 20.0 days (percentile 25 to percentile 75, 11.0 to 30.0 days).

### Outcomes

Nineteen (19.0%) patients underwent CSF shunt placement due to hydrocephalus. In-hospital mortality was observed in 18 (18.0%) patients. Persistent neurological sequelae among survivors included walking difficulty in eight cases, visual impairments in three, including one patient with blindness, speech difficulty in three, and fecal and urinary incontinence in one.

**Fig. 1:**
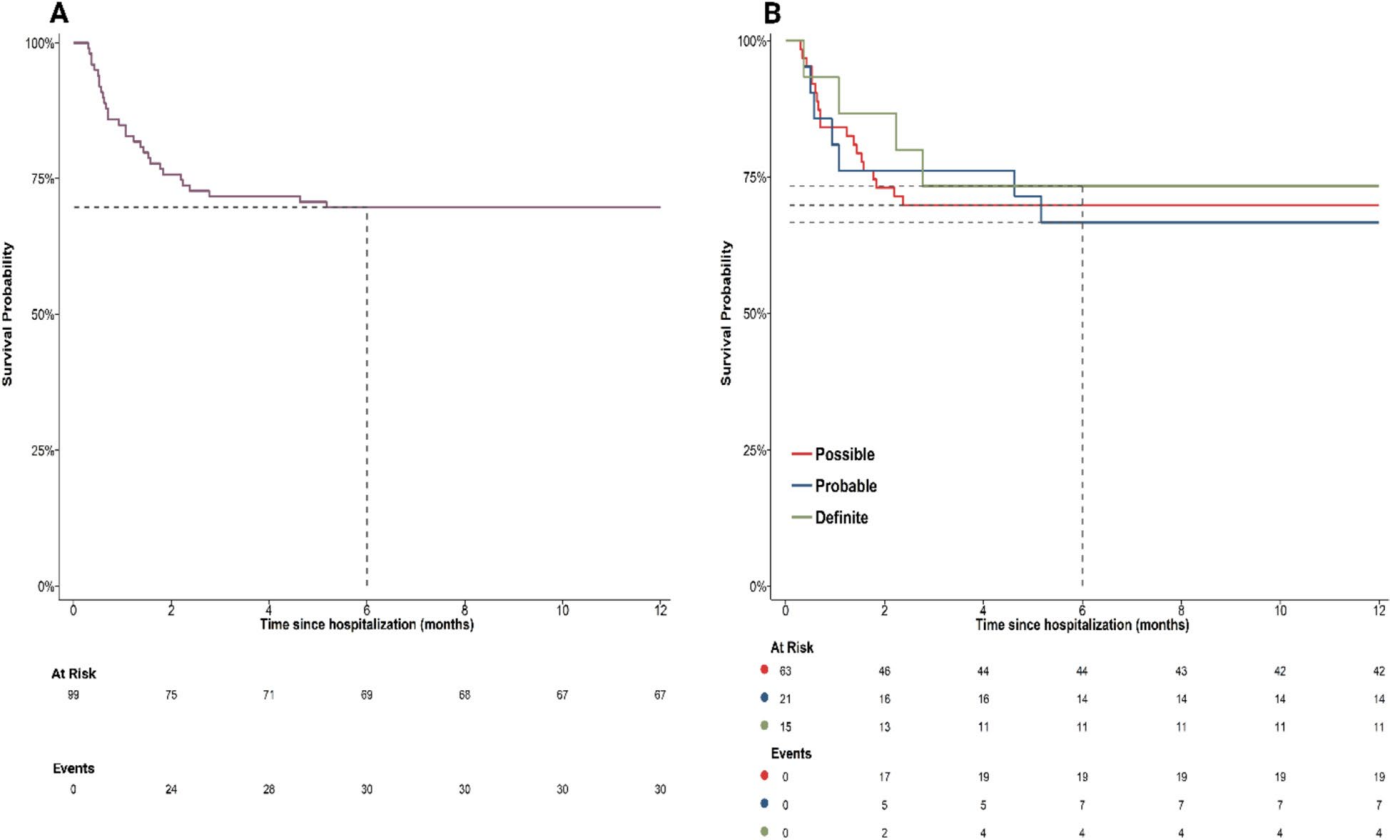
Kaplan–Meier survival curves for patients with tuberculous meningitis: (**A**) for the entire population, and (**B**) stratified by the certainty of diagnosis. The X-axis indicates time (in months), while the Y-axis shows cumulative survival probability. Dashed lines represent the survival rates at 6 months

By the end of 6 months, the survival rate of included patients was 69.7% (95% CI 59.6% to 77.7%) (Fig. 1A). The 6-month survival rates, based on the certainty of diagnosis, were 69.8% (95% CI 56.9%–79.6%) for a possible diagnosis, 66.7% (95% CI 42.5%–82.5%) for a probable diagnosis, and 73.3% (95% CI 43.6%–89.1%) for a definite diagnosis (Fig. 1B). The rate after 12 months was similar to that after 6 months.

There was no significant difference between possible, probable, and definite diagnoses in terms of 12-month mortality (Log-rank [Mantel–Haenszel estimate] test *P*-values for comparison of possible vs. probable diagnosis: 0.797; possible vs. definite diagnosis: 0.728; and probable vs. definite diagnosis: 0.662).

A significant difference was observed between different stages of BMRC in terms of survival (Log-rank [Mantel–Haenszel estimate] test P-values for the comparison of stage I vs. stage II: 0.055; stage I vs. stage III: 0.001; and stage II vs. stage III: 0.021). In addition, the *P*-value for the linear trend was also significant across stages of BMRC (*P*-value < 0.001).

**Fig. 2:**
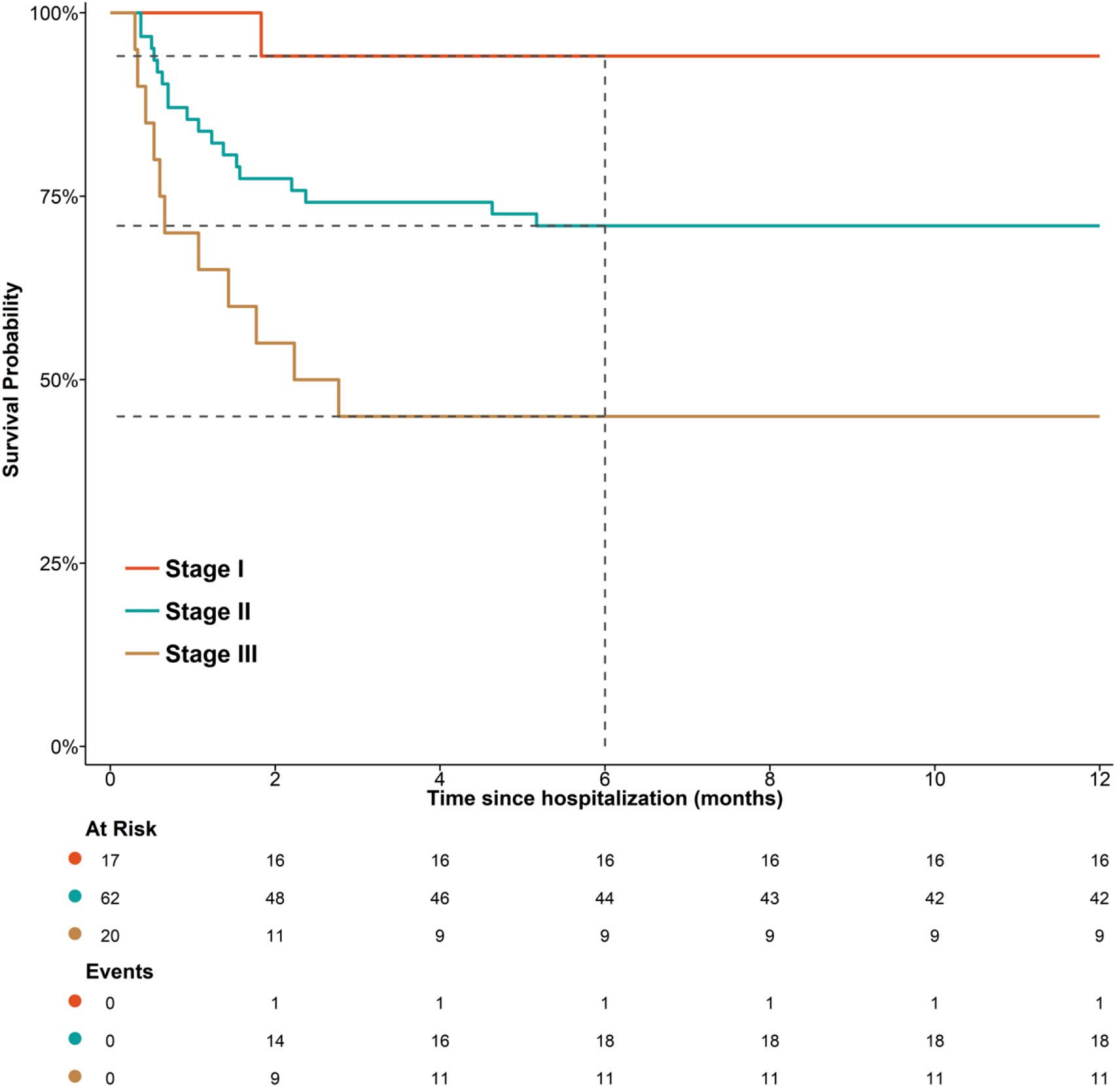
Kaplan–Meier survival curves for patients with tuberculous meningitis, stratified by the stage of BMRC. The X-axis indicates time (in months), while the Y-axis shows cumulative survival probability. Dashed lines represent the survival rates at 6 months. BMRC, British Medical Research Council

Stage II (hazard ratio [HR]: 5.69, 95% CI 0.76 to 42.65, *P*-value: 0.091) compared to stage I showed a marginal increase in mortality, while stage III (HR: 13.51, 95% CI 1.74 to 104.76, *P*-value: 0.013) compared to stage I revealed a significantly elevated risk of mortality during the 12-month period. Stage III compared to stage II showed a 2.37-fold increase in the mortality risk throughout the study period (HR: 2.37, 95% CI 1.12 to 5.03, P-value: 0.024) (Fig. 2).

### Predictors of mortality

Univariable analysis of continuous variables showed that age (HR: 1.04, 95% CI 1.02 to 1.06, P-value: < 0.001), hemoglobin level (HR: 0.72, 95% CI 0.61 to 0.85, P-value: < 0.001), and erythrocyte sedimentation rate (ESR) (HR: 1.02, 95% CI 1.01 to 1.03, P-value: 0.001) were significantly associated with mortality (Table 3). After adjusting for age, the results for ESR and hemoglobin remained significant.

**Table 3:** Predictors of mortality based on an increase in the unit of variable

Variable	Univariable analysis		Multi-variable analysis	
	Hazard ratio (95% CI)	*P-value*	Hazard ratio (95% CI)	*P-value*
Age (years)	1.04 (1.02 to 1.06)	< 0.001	–	–
Peripheral leukocytes (× 10^3^/per µl)	1.02 (0.95 to 1.10)	0.540	1.04 (0.97 to 1.13)	0.268
Hemoglobin (mg/dL)	0.72 (0.61 to 0.85)	< 0.001	0.75 (0.64 to 0.89)	0.001
Platelets (× 10^3^/per µl)	1.00 (1.00 to 1.00)	0.180	1.00 (1.00 to 1.01)	0.242
ESR (mm/hour)	1.02 (1.01 to 1.03)	0.001	1.02 (1.01 to 1.04)	< 0.001
CSF leukocyte count (per µl)	1.00 (1.00 to 1.00)	0.650	1.00 (1.00 to 1.00)	0.759
CSF protein level (mg/dl)	1.00 (1.00 to 1.01)	0.977	1.00 (1.00 to 1.00)	0.906
CSF glucose level (mg/dl)	1.00 (0.98 to 1.01)	0.829	1.00 (0.98 to 1.01)	0.610

CI, confidence interval; HR, hazard ratio; ESR, erythrocyte sedimentation rate; CSF, cerebrospinal fluid

**Fig. 3:**
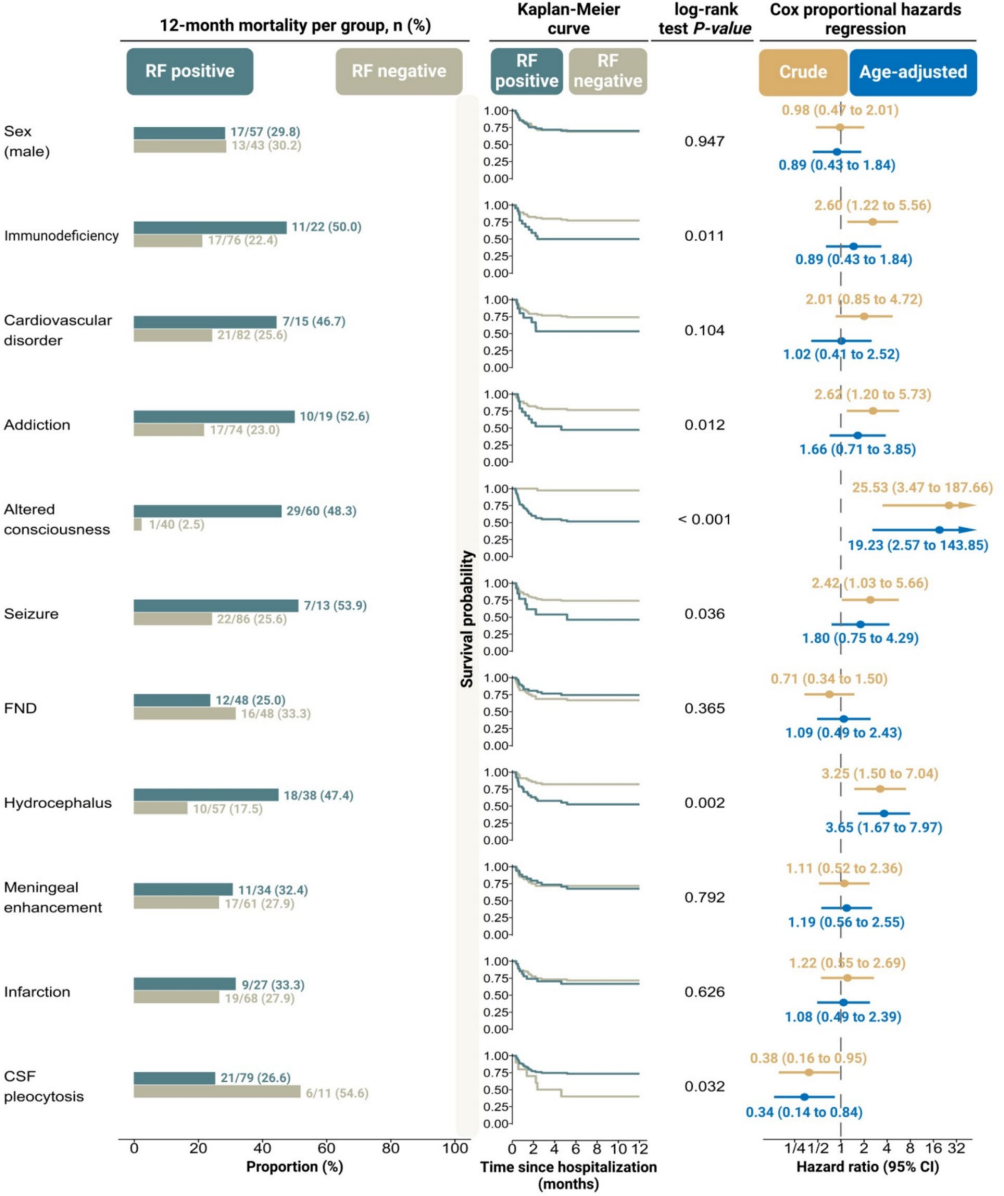
Analysis of univariable and age-adjusted models examining the relationship between different clinical and neuroimaging features and the 12-month mortality risk in patients with tuberculous meningitis. HR, hazard ratio; CI, confidence interval; RF, risk factor; FND, focal neurologic deficit; CSF, cerebrospinal fluid

In the crude model assessing binary potential predictors of 12-month mortality, immunodeficiency (HR: 2.60, 95% CI 1.22 to 5.56), substance addiction (HR: 2.62, 95% CI 1.20 to 5.73), altered consciousness (HR: 25.53, 95% CI 3.47 to 187.66), and hydrocephalus (HR: 3.25, 95% CI 1.50 to 7.04) were significantly associated with an increased mortality rate during the 12-month follow-up period (Fig. 3).

In addition, CSF pleocytosis (HR: 0.38, 95% CI 0.16 to 0.95) was negatively linked to mortality at the end of 12 months. Regarding the age-adjusted model, only the association of altered consciousness (HR: 19.23, 95% CI 2.57 to 143.85), hydrocephalus (HR: 3.65, 95% CI 1.67 to 7.97), and CSF pleocytosis (HR: 0.34, 95% CI 0.14 to 0.84) with 12-month mortality remained significant.

## Discussion

The present study provides comprehensive insights into the clinical characteristics, neuroimaging findings, laboratory features, diagnosis, and outcomes of patients with TBM. Notably, the in-hospital mortality rate was 18%, with a survival probability of 69.7% at 12 months. Persistent neurological sequelae among survivors included walking difficulty, visual impairments, speech difficulty, and fecal and urinary incontinence. Approximately, twenty percent of patients required the placement of a CSF shunt.

Our study contributes to understanding the complexities of managing TBM, particularly in contexts where diagnostic tests are hindered by their suboptimal sensitivity and time-consuming nature, as extensively documented in the literature [17]. These challenges underscore the difficulties in achieving timely diagnosis and initiating prompt treatment, which are crucial for improving patient outcomes in TBM. Our study utilized the classification criteria proposed by Marais et al. in the *Lancet Infectious Disease* [12] to assess the diagnostic certainty of TBM, with 63.0% of cases classified as possible, 22.0% as probable, and only 15.0% as definite TBM. It demonstrates that under our standard treatment approach, which included not only definite TBM cases but also possible and probable cases after excluding alternative diagnoses (as described in the Methods), the outcomes of patients with TBM were comparable across these diagnostic categories. This approach helps mitigate the risks of delayed treatment. Another retrospective cohort study of 41 Mexican adults reported a 19.5% death rate, with no statistically significant difference in mortality between those with and without a definitive diagnosis (*P*-value = 0.109), further supporting our findings [2]. On the other hand, when CSF positivity for tubercle bacilli is used as the sole criterion for classifying TBM as a definite diagnosis, studies have indicated that patients with detectable tubercle bacilli in the CSF often experience poorer outcomes compared to those with a possible diagnosis. This highlights the impact of a higher bacterial burden, with research showing worse prognoses and higher rates of severe disturbances in consciousness in patients with positive CSF tests for tubercle bacilli [18–20].

In our cohort, the 12-month survival probability was 69.7%. In contrast, studies from high-income countries reported better outcomes: for instance, a Spanish cohort reported 81.8% survival at 12 months with 28.3% experiencing sequelae [21], and a U.S. multi-state cohort of 806 TBM patients reported 18.3% mortality at 12 months, alongside complications like stroke (15%), seizures (11.9%), and vision impairment (18.7%) [22], with an in-hospital mortality rate of 10.5% compared to 18% in our study. Mortality rates in low- and middle-income countries (LMICs) are more variable and often higher, ranging from 4.4 to 38.9%, as reported in China [18, 23, 24], Ecuador [25], India [26–28], Pakistan [29], and Madagascar [30]. A 2019 meta-analysis further confirmed a global mortality risk of 24.7% and a 50.9% rate of neurological sequelae among survivors [31]. Additionally, the New York cohort demonstrated that more than half of TBM patients died before completing antituberculosis therapy, with most deaths occurring within the first month—highlighting that mortality measured only during treatment may underestimate the true burden of TBM-related deaths [5].

Our study was conducted in a resource-limited middle-income country, and our findings highlight diagnostic and management challenges typical of such settings, particularly the difficulty in confirming TBM microbiologically. This reflects broader inequities in neurological care, as emphasized by Uwishema & Boon [32], who underscored how gaps in access to diagnostics, trained healthcare personnel, and infrastructure disproportionately affect patient outcomes in LMICs. In line with their recommendations, improving access to diagnostic tools, enhancing training, and reducing healthcare inequities could significantly improve TBM outcomes in similar settings.

In our study, we identified several significant predictors of mortality in TBM patients, particularly the presence of hydrocephalus and altered consciousness at presentation. These findings highlight the increased vulnerability of individuals with hydrocephalus and emphasize the critical need for early detection and intervention in cases presenting with altered mental status. Moreover, a higher ESR and older age were associated with an increased risk of mortality, underscoring both age and ESR as potential indicators of TBM severity. Hemoglobin levels and the presence of CSF pleocytosis also emerged as significant factors, suggesting that these may be negatively correlated with mortality in TBM.

Other studies have identified additional predictors of mortality in TBM. A study from New York found initial drug resistance and HIV infection to be powerful predictors of all-cause mortality over a ten-year period [5]. A 2019 meta-analysis reported significantly higher mortality rates during treatment in patients diagnosed at stage III of TBM or those who were HIV-positive, with mortality rates of 64.8% and 53.4%, respectively [31]. Another study from Southern Vietnam, which included 1,699 patients, developed prognostic models for 9-month mortality in adults with TBM, identifying higher BMRC disease severity and lower CSF lymphocyte counts as key predictors. For HIV-uninfected patients, older age and previous tuberculosis were additional risk factors, while lower weight and CD4 cell count were significant for HIV-infected patients [33].

In our cohort, altered consciousness was a strong predictor of mortality; however, the analysis should be interpreted with caution due to the sparse data (mortality rate) in patients with normal consciousness (n = 1, 2.5%). The wide confidence interval observed for the hazard ratio of altered consciousness (HR: 19.23, 95% CI 2.57–143.85) suggests some degree of uncertainty, likely due to the small number of events in this category. However, the consistency of altered consciousness as a significant predictor across multiple studies reinforces its clinical importance in TBM prognosis. The higher stages, based on the modified BMRC disease severity grading, were significantly associated with higher mortality. Furthermore, a linear trend was noted across the stages, highlighting a possible dose–response relationship. This is in line with most studies, which have identified BMRC grading as a robust independent predictor of outcomes [34]. Additionally, most previous studies have identified hydrocephalus as an independent predictor of mortality [18, 23, 25–27, 29, 34]. Increased intracranial pressure, primarily due to hydrocephalus, is common in TBM patients and, if untreated, can exacerbate cerebral ischemia caused by perfusion-limiting vasculitis—a hallmark of TBM.

Our study found that higher CSF pleocytosis was inversely associated with mortality in TBM patients, a finding that aligns with previous research. Specifically, a study from Vietnam reported that low CSF leukocyte counts—particularly low neutrophil counts—were linked to increased mortality, along with low glucose and high lactate levels [35]. Similarly, in bacterial meningitis, low CSF white blood cell counts have been identified as strong predictors of poor outcomes. In a large prospective cohort study of adults with community-acquired bacterial meningitis, van de Beek et al. reported that a low CSF leukocytes (< 1000/mm^3^) was independently associated with an unfavorable outcome [36]. It is hypothesized that a low CSF leukocyte response may reflect an impaired immune response or overwhelming infection, both contributing to increased mortality risk. This mechanism could also apply to TBM, where a diminished CSF pleocytosis might indicate inadequate immune activation against *M. tuberculosis*, resulting in a more severe disease course [36]. While elevated CSF inflammatory cytokines, such as IL-6, have been associated with severe disease at presentation, they did not correlate with death or disability in HIV-negative TBM patients [6]. These findings suggest that CSF pleocytosis may serve as a useful prognostic marker, reflecting the host’s ability to mount an effective immune response in CNS infections. Further studies are warranted to better elucidate the underlying immunopathological mechanisms.

The rate of HIV infection in our cohort was remarkably low at 1.0%, which is notable given the high prevalence of HIV in most other neurotuberculosis studies. In a systematic review of HIV-TBM co-infection, studies published between January 1, 2000, and January 31, 2017, were analyzed to estimate the prevalence of co-infection across 26 studies. The prevalence of HIV among patients with confirmed TBM was 30% (95% CI 12 to 47), while it was 12.1% (95% CI 7.3 to 19.2) among those with suspected TBM. These findings highlight a considerable prevalence of TBM-HIV co-infection [37]. This discrepancy may be explained by the local epidemiology of HIV in Mashhad and Khorasan Razavi province. According to the Health Deputy of Mashhad University of Medical Sciences, the estimated number of people living with HIV in Khorasan Razavi is 1,063, of whom approximately 50% have been officially identified. Considering that the population of Khorasan Razavi province was over 6.4 million based on the 2016 national census, the overall HIV prevalence in the region remains relatively low compared to high HIV burden settings. This epidemiological context likely contributes to the low rate of HIV-TBM co-infection observed in our cohort. Furthermore, the provision of antiretroviral therapy to 95% of identified cases, along with accessible screening services, may further reduce the risk of HIV-associated TBM in this population [38].

Given that HIV significantly contributes to neurological disorders, including TBM, especially in advanced stages of immunosuppression, understanding this epidemiological context is crucial [32]. Importantly, despite advancements in antiretroviral therapy, HIV-infected individuals continue to face substantial risks of neurological complications, emphasizing the need for vigilance in settings with high HIV prevalence.

Fever and headache were the most prevalent symptoms, occurring in 64.7% and 63.6% of our patients, respectively. These findings align with the literature, where common symptoms of TBM include headache, fever, and vomiting [39]. Altered consciousness was observed in 60.0% of cases, while focal neurological deficits were present in 50%. Notably, the classic triad of meningitis—fever, altered consciousness, and neck stiffness—was observed in only 18% of patients. In our study, 27% of patients with TBM developed cranial neuropathy, with approximately 46% of these cases involving multiple cranial nerves, primarily affecting the abducent, oculomotor, and facial nerves. Cranial nerve involvement in TBM often results from vascular compromise, ischemia, or nerve entrapment within basal exudates [40]. Previous research indicated that older age, altered sensorium, severe functional disability, and specific CSF abnormalities—such as elevated protein levels and high CSF leukocytes—being significant predictors for cranial neuropathy in TBM [41]. Another South China study found a 33.3% prevalence of cranial nerve palsy, with neurological deficits, extracranial TB, and elevated CSF leukocyte count as key risk factors [42]. A large study reported that 14.8% of 486 TBM patients had cranial nerve palsy, with the oculomotor and optic nerves being most affected. Nevertheless, 97.2% of patients with cranial nerve palsy fully recovered with two months of anti-TB treatment [43].

CSF analysis in our study revealed pleocytosis in 87.8% of patients, predominantly lymphocytic (70.6%). Hypoglycorrhachia was observed in 64.0% of cases, and hyperproteinorrhachia in 91.0%. As noted in previous studies and confirmed in our findings, CSF leukocyte counts can be normal in TBM, especially among individuals with compromised cell-mediated immunity, such as the elderly or those with HIV. Neutrophils may be predominant in the early stages of the disease. Prior research indicates that a higher proportion of neutrophils in the CSF is associated with a greater likelihood of bacteriological confirmation and improved survival rates [6].

In our study, neuroimaging revealed hydrocephalus in 40.0% and meningeal enhancement in 35.8% of TBM patients. Higher rates of meningeal enhancement, up to 90%, have also been reported in previous studies [40]. Cerebral infarctions were observed in 28.4% of patients, aligning with the 20–41% range documented in prior research [40]. These infarctions predominantly occur in the basal ganglia or internal capsule regions due to necrotizing arteritis from small vessel occlusion [40]. The areas supplied by the proximal middle cerebral artery and the medial lenticulostriate and thalamoperforating vessels, often referred to as the medial tubercular zone, are particularly vulnerable to such vascular involvement [34]. Hydrocephalus was observed in a substantial portion of our cohort, reflecting common obstruction of CSF flow in TBM patients. The variability in imaging findings, such as tuberculomas and meningeal enhancement, highlights differences across studies, with rates of hydrocephalus ranging from 26 to 82% and meningeal enhancement from 30 to 79% [18, 23, 24, 26, 27, 29, 30, 44]. Additionally, about 6% of our patients presented with intracranial tuberculous mass lesions, including one notable pregnant woman with a large cerebellar mass initially suspected to be neoplastic. Histopathological examination of the resected brain mass confirmed granulomatous inflammation with caseous necrosis, highlighting the importance of considering TBM in the differential diagnosis of intracranial mass lesions, especially in regions with high TB prevalence. Diagnosing these lesions often relies on radiological features, evidence of TB elsewhere, and treatment response. However, responses can be unpredictable, with lesions sometimes enlarging or persisting despite appropriate therapy [45].

Our study, which utilized the diagnostic criteria proposed by Marais et al. (2010), demonstrated substantial variability in diagnostic certainty, with 63.0% of cases classified as possible, 22.0% as probable, and only 15.0% as definite TBM. In our study, a definite diagnosis of TBM was achieved in only a small percentage of patients, reflecting the ongoing difficulties in microbiological confirmation of TBM. Traditional methods like acid-fast smear microscopy have limited sensitivity, especially in cases with low mycobacterial counts. Newer techniques, such as fluorescent microscopy, Nucleic Acid Amplification Test (NAATs), and the Xpert MTB/RIF, offer improved diagnostic capabilities with higher specificity, though sensitivity remains variable. While the Xpert MTB/RIF has shown promise, particularly in high-burden regions, concerns about false positives in areas with low rifampicin resistance prevalence highlight the need for more reliable and consistent diagnostic approaches in TBM [46]. Although our study primarily relied on AFB smear, culture, and CSF PCR for microbiological confirmation, previous studies have evaluated additional non-culture-based diagnostic tools, such as adenosine deaminase (ADA), IGRA, and Gene Xpert, reporting varying sensitivities. ADA has limited diagnostic value for TBM due to low specificity and variability [46]. Combining non-culture tests such as ADA, IGRA, and CSF PCR may improve early TBM diagnosis, particularly in resource-limited settings [47].

Our study has several limitations. First, although part of the data was collected prospectively, a significant portion was obtained retrospectively. The retrospective component may have introduced selection and information biases, potentially affecting data completeness and accuracy. Second, while we utilized the Marais et al. criteria for TBM classification, the reliance on clinical and laboratory parameters for diagnosing possible and probable TBM cases, without microbiological confirmation, raises the risk of misclassification. Although our treatment approach included all patients meeting these criteria after ruling out alternative diagnoses, variations in diagnostic certainty may have influenced outcome comparisons. Third, the low proportion of definite TBM cases (15%) reflects the ongoing challenges of microbiological confirmation in resource-limited settings, where diagnostic tools such as NAATs or mycobacterial cultures are often not readily available. While this approach reflects real-world clinical practice in similar settings, it may limit the generalizability of our findings to settings with greater diagnostic capacity, where a higher proportion of microbiologically confirmed cases could influence both case management strategies and outcomes. Furthermore, the inclusion of probable and possible cases may introduce heterogeneity, although it also underscores the need for empirical treatment in contexts where delays in diagnosis can be fatal. Fourth, telephone-based follow-up for assessing survival and neuropsychosocial outcomes, while practical, may introduce recall and response bias. To mitigate this, we employed structured interviews conducted by trained personnel using standardized checklist, with caregiver input when available. However, the lack of in-person assessments remains a limitation. Additionally, detailed information on treatment regimens, including corticosteroid dosing, duration, tapering schedules, and the use of second-line therapies, was not consistently available, particularly after hospital discharge. While all patients were initiated on standard four-drug anti-TB therapy for 12 months and the majority received adjunctive corticosteroids during hospitalization, variability in post-discharge management limits our ability to analyze the impact of specific treatment strategies on outcomes. Fifth, detailed information regarding the specific causes of death was not consistently available. As our study focused on reporting all-cause and long-term mortality, distinguishing between TBM-related and unrelated causes of death was not possible. This may limit deeper interpretation of mortality patterns in our cohort. Lastly, the notably low prevalence of HIV in our cohort, compared to global TBM studies, may restrict the generalizability of our results to regions with a higher burden of TB-HIV co-infection. Future prospective studies with larger sample sizes, broader diagnostic confirmation, and objective neuropsychosocial assessments are needed to validate and expand upon our findings.

## Conclusion

This study highlights the high mortality and morbidity associated with TBM and reinforces the critical importance of timely diagnosis and intervention. Our findings demonstrate comparable survival rates across definite, probable, and possible TBM categories, supporting the value of empirical treatment, particularly in resource-limited settings where diagnostic confirmation is often challenging. Improving access to essential interventions, such as CSF shunt placement, and enhancing the availability of rapid, sensitive diagnostic tools may help reduce mortality and long-term neurological sequelae. Future research should focus on refining diagnostic strategies and optimizing care pathways to further improve outcomes.

## Data Availability

The data underlying this article will be shared upon reasonable request to the corresponding author. No datasets were generated or analysed during the current study.

## Abbreviations

TBM: Tuberculous meningitis
CSF: Cerebrospinal fluid
NAAT: Nucleic acid amplification test
HR: Hazard ratio
CI: Confidence interval
BMRC: British Medical Research Council
ESR: Erythrocyte sedimentation rate
CNS: Central nervous system
GCS: Glasgow Coma Scale
ADA: Adenosine deaminase
ZN: Ziehl Neelsen
ACS: Automated culture system
LJ: Lowenstein Jensen
